# Composite Magnetic Sorbents Based on Iron Oxides in Different Polymer Matrices: Comparison and Application for Removal of Strontium

**DOI:** 10.3390/biomimetics5020022

**Published:** 2020-05-18

**Authors:** Andrei Egorin, Eduard Tokar, Anna Matskevich, Nikita Ivanov, Ivan Tkachenko, Tatiana Sokolnitskaya, Larisa Zemskova

**Affiliations:** Institute of Chemistry, Far Eastern Branch, Russian Academy of Sciences, 690022 Vladivostok, Russia; d.edd@mail.ru (E.T.); mysmatskevich@mail.ru (A.M.); ivanov.np@students.dvfu.ru (N.I.); tkachenko@ich.dvo.ru (I.T.); sokolnitskaya@ich.dvo.ru (T.S.); zemskova@ich.dvo.ru (L.Z.)

**Keywords:** magnetic chitosan composites, polymeric sorbents and biosorbents, iron oxides, strontium, adsorption

## Abstract

Introduction of magnetic nanoparticles into composite sorbents based on polymer matrices has received great attention due to the possibility of using cheap iron oxides and removing spent sorbents by means of magnetic separation. In the present paper, we discuss the problem of creating magnetic sorbents using two types of matrices as host materials: synthetic cation exchange resin and natural aminopolysaccharide chitosan. The possibilities of applying matrices for the in situ formation of oxide phases of a specified composition with the required content of an inorganic component in a composite material were estimated. The composition of the oxide phase formed in the composite material was studied, and particle sizes were evaluated by the method of X-ray diffraction analysis. Magnetic characteristics were investigated. Sorption characteristics with respect to strontium for the composites containing iron oxides were determined.

## 1. Introduction

To solve many problems of environmental protection, sorption methods employing various sorbents are used extensively; the popular sorbents, aside from carbon materials and ion-exchange resins, include those based on natural biopolymers, for example, cellulose and its modifications [1,2,3,4,5,6] or chitosan [7,8,9,10,11,12,13,14].

In recent years, numerous nanosized metal oxides/hydroxides, such as those of iron, aluminum, manganese, titanium, magnesium, cerium, etc., have received ever-increasing attention of researchers as adsorbents [15,16]. They can be used as catalytic materials, coloring agents, gas sensors, magnetic resonance imaging, and drug delivery agents. The most promising of these metal oxides in the function of adsorbents are iron oxides and composites based on them, which can be used for the removal of heavy metals, radionuclides, and arsenic, as well as for the isolation and concentration of organic compounds [17,18,19]. Iron oxides are cheap, non-toxic, and have a wide range of applications, but their main advantage consists in the fact that they can be removed from the solutions to be decontaminated by magnetic separation [17,20].

Numerous researchers reported that there were a number of problems to be solved in order to apply nanopowders directly in decontamination systems. First, oxide nanopowders are normally unstable and tend to agglomerate. Second, recycling powders from treated waters (except for magnetic nanomaterials) is practically impossible due to their incomplete separation and contamination of the treated solutions with nanoparticles, the toxicity of which has not been exhaustively determined [15,16,17,20].

To overcome these technical difficulties, iron oxide nanopowders were introduced to conventional porous adsorbents, including activated carbon, cellulose and alginate granules, and synthetic resins [16,19,21]. Both cation- and anion-exchange materials can be used as synthetic resins [21]. The application of a macroporous polystyrene anion exchanger, D-201, was reported earlier [22]. The authors of [23] investigated magnetic materials based on microporous and biporous hypercrosslinked polystyrenes and a mesoporous sorbent, XAD-4. The authors of [24] compared the sorption properties with respect to arsenic for two hybrid sorbents; the first one contained hydrous iron oxides and was fabricated on the basis of a strongly basic gel-type anion exchanger, a resin with quaternary amine functional groups, and an ArsenX^np^ sorbent. The commercial sorbent ArsenX^np^ is a hybrid sorbent in which nanosized iron oxide particles are introduced into a strong-base anion exchanger based on a polystyrene matrix with quaternary amino groups [21,25].

However, ion-exchange resins are characterized by high cost, and their synthesis technologies are rather complicated; furthermore, the raw materials for their production are not renewable. Natural biopolymers can serve as alternatives to ion-exchange resins; the comparison characteristics of synthetic polymer matrices and biopolymers were provided in [16]. The former ones were characterized by a cross-linked structure suitable for stable immobilization, excellent mechanical properties, chemical stability and compatibility, regulated structure, a charged surface that promotes the dispersion of the modifier and pollutant nanoparticles, and easiness of separation from solutions. On the other hand, biopolymers are readily available and widely spread, are built from moderately flexible chiral molecules, are hydrophilic, and are environmentally safe.

Chitosan is the most favorable among the biopolymers for the immobilization of inorganic components or “guests”, which was stated in reviews [16,17,18,19,20,21,26] and articles [27,28,29,30]. A natural polysaccharide chitosan is a product of deacetylation of chitin and characterized with a unique combination of properties, such as biodegradability, bioactivity, biocompatibility and non-toxicity, and low ash content. However, it has also significant disadvantages, including low stability in acidic media, unsatisfactory mechanical properties, low thermal stability, resistance to mass transfer, and low porosity and surface area. These disadvantages can be overcome by preparing chitosan composites or by modifying chitosan using physical or chemical methods. Physical modification is performed by converting the chitosan powder into a gel (granules, membranes, films, etc.) or nanoparticles. This can result in the increase of the porosity, surface area, and number of adsorption sites, the reduction of crystallinity, and the increase of swelling and diffusion characteristics. Chemical modification can increase the stability and reduce the solubility in an acidic medium. It includes cross-linking of polymer units, grafting selective groups on the polymer chain, and impregnation.

The functionalized polymer phase mainly serves as a host, while inorganic particles of the metal oxide phase are finely dispersed in the host material. Synthesis of such hybrid materials can be carried out in two ways: (1) by dispersing ready-made iron oxide nanoparticles in a preliminarily obtained or commercial polymer; (2) and directly through the process of preparation (or deposition) of the polymer [20,21].

Iron oxides are adsorbents for various classes of contaminants; however, using such inorganic ion exchangers for the removal of radionuclides is of urgent interest. In the present work, we describe the results of strontium removal by two types of hybrid sorbents based on iron oxides immobilized into different inorganic matrices (ion-exchange resin and chitosan). The works on the application of iron oxides for removal of the Sr-90 isotope are not numerous and describe as natural oxide minerals as synthetic oxides of various compositions embedded into a chitosan matrix. Besides, it is important to determine what type is necessary to select for synthesis of an active component in it, namely, iron oxide, since the biopolymer, unlike the ion-exchange resin, is known to be incapable of adsorbing strontium.

Strontium represents one of the relevant contaminants in terms of radioecological safety. As was reported earlier, iron oxide minerals [31] and synthetic iron oxides of various compositions immobilized in a chitosan matrix [30,32,33,34] were used for the removal of strontium.

It appears urgent to compare the capabilities of composite materials in which the active component (iron oxide) is immobilized in a polymer matrix during its production in situ. The objective of the present work was to prepare magnetic sorbents based on a strong KU-2 cation exchanger (styrene and divinylbenzene copolymer) and a natural polysaccharide chitosan to study the composition of the formed iron oxide phases, evaluate the size of particles formed in polymer matrices, and determine and compare the magnetic characteristics and sorption properties of the resulting composites with respect to strontium.

## 2. Results

### 2.1. Sorbents Characteristics

**Figure 1:**
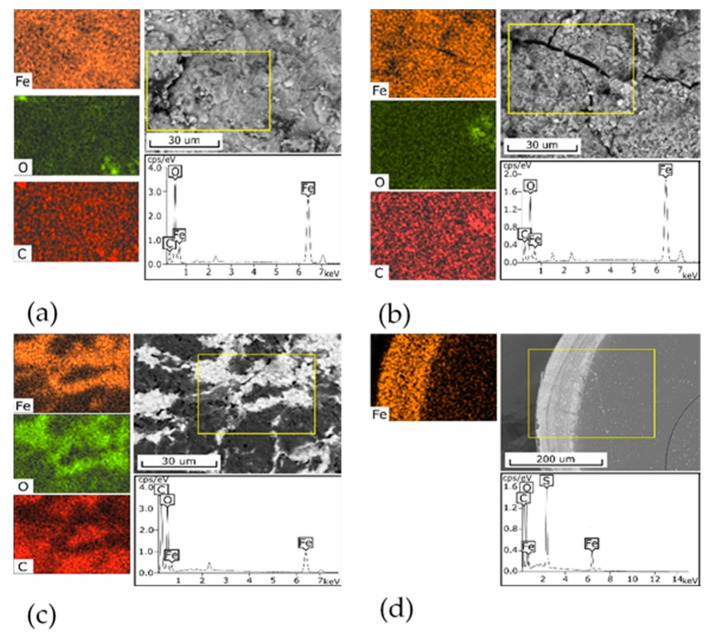
SEM-EDX analysis of composite sorbents, the yellow rectangle is the analysis area: (**a**) 3M, (**b**) 3M-2, (**c**) 3M-4, (**d**) KU-MAG. Scale bar (**a**–**c**) 30 μm, (**d**) 200 μm.

Figure 1 shows the SEM-images of the surface of the 3M, 3M-2, and 3M-4 sorbents (Figure 1a–c) and the cross-section of the KU-MAG sorbent granule (Figure 1d). The surface of the 3M sorbent is monolithic, with inclusions of flakes and particles formed, probably, in the process of the material grinding. The iron distribution over the surface is relatively homogeneous. The surface of the 3M-2 is looser while the iron distribution is also relatively homogeneous. The morphology of the 3M-4 sorbent is heterogeneous, has a sponge-like structure, and contains areas different in color. According to the element distribution map, light areas are, probably, represented by an iron oxide phase, whereas dark areas correspond to chitosan. The cross-section of a KU-MAG particle is even, the upper layer, with a thickness of 70–80 μm, is lighter and is represented by an iron oxide phase in accordance with the element distribution map.

**Figure 2:**
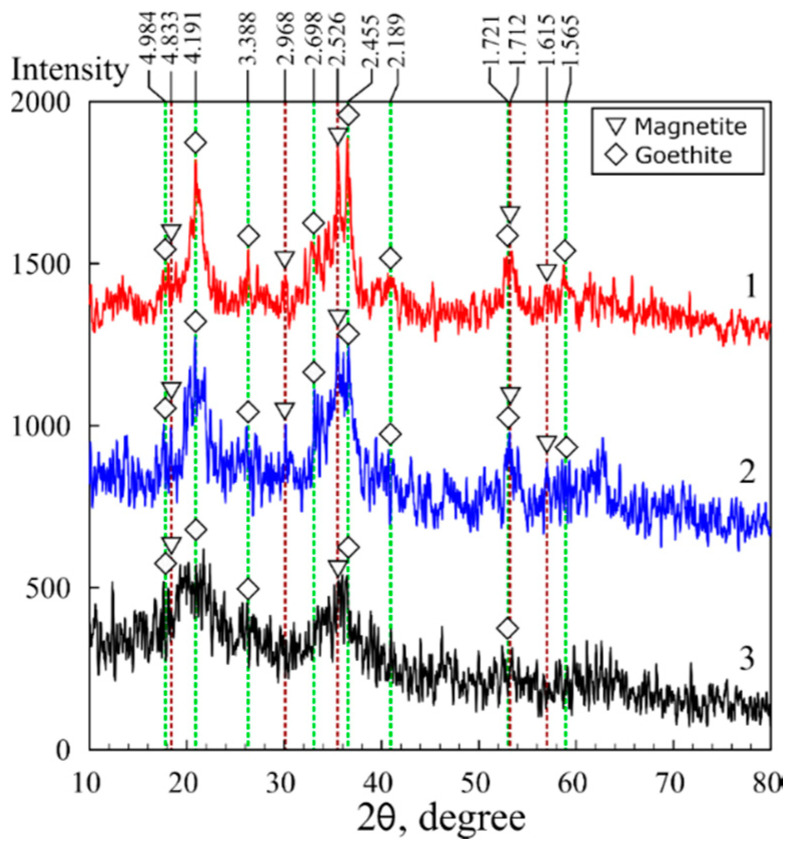
X-ray diffraction patterns of the magnetic composites based on chitosan: 1—sorbent 3M, 2—sorbent 3M-2, 3—sorbent 3M-4.

**Figure 3:**
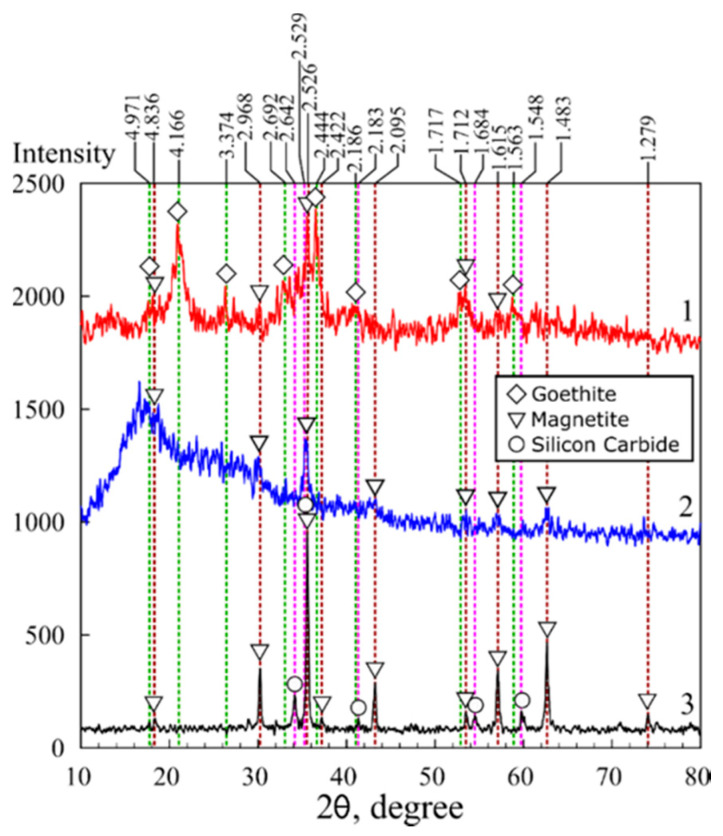
X-ray diffraction patterns: 1—sorbent 3M, 2—sorbent KU-MAG, 3—sorbent MAG.

The results of the X-ray diffraction analysis of the magnetic sorbents in polymer matrices are shown in Figure 2 and Figure 3. Fe_3_O_4_ magnetite (00-900-2674) and FeO(OH) goethite (00-901-0406) were identified in the chitosan matrix using the Crystallography Open Database (COD) [35]. Moreover, the MAG sample data were characterized by the presence of peaks attributed to the phase of silicon carbide (00-101-1053), likely related to impurities in the initial chitosan.

Crystalline sizes estimated on the basis of the XRD data of the prepared composites for different 2θ are presented in Table 1.

Table 2 shows the results of estimation of the stability of composite sorbents in an NaCl solution of a concentration of 0.1 mol/L. In accordance with the presented data, one can conclude that composite sorbents are distinguished with high mechanical stability and do not tend to destruct with prolonged contact with the solution to be decontaminated.

### 2.2. Magnetic Properties

**Figure 4:**
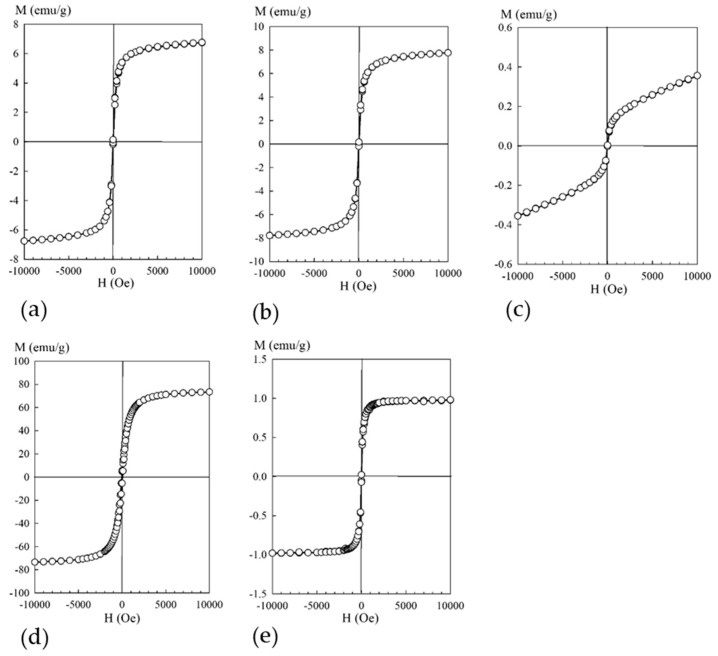
Dependence of the magnetization M (emu/g) on the magnetic field H (Oe): (**a**) sorbent 3M, (**b**) sorbent 3M-2, (**c**) sorbent 3M-4, (**d**) sorbent MAG, (**e**) sorbent KU-MAG.

Figure 4 shows field dependences of the magnetization of the composites with different contents of iron oxide (obtained at room temperature).

### 2.3. Sorption Properties

**Figure 5:**
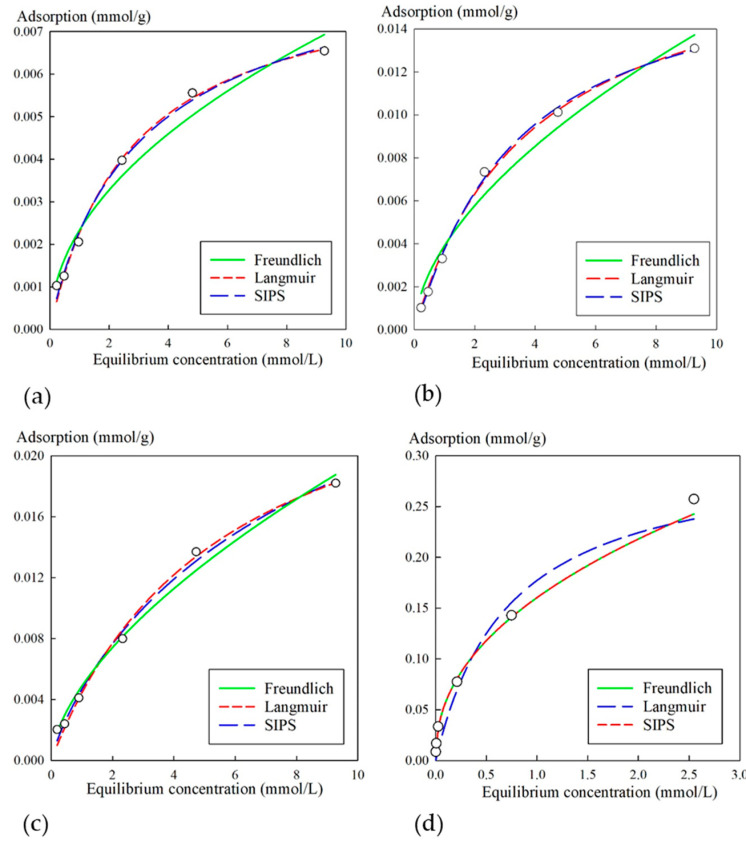
Isotherms of Sr^2+^ sorption from NaCl solution (0.1 mol/L) for sorbents: (**a**) 3M, (**b**) 3M-2, (**c**) 3M-4, (**d**) KU-MAG. Circles represent the experimental data; fit lines were computed using the Freundlich, Langmuir, and Sips equations (Equations (2), (3), and (4), respectively).

Figure 5 shows isotherms of strontium sorption from a neutral NaCl solution of a concentration of 0.1 mol/L and nonlinear regression curves obtained using respective equations. The obtained sorption isotherm according to Giles classification could be attributed to the L-type or H-type, which indicated high affinity of the composite materials with respect to Sr^2+^ ions in the region of low concentrations.

Table 3 shows the calculated parameters for experimental values using their respective equations. The KU-MAG sorbent was characterized with the highest values of *G_max_*, as well as *K_F_* and *K_L_*; here, the experimental values could not be adequately described by the Sips equation, in spite of high correlation coefficients. However, it is worth mentioning that high sorption characteristics resulted from adsorption on functional groups of the cation-exchanger rather than on the iron oxide phase. Specific features of the strontium sorption on modified cation-exchangers were discussed in detail in [36].

**Figure 6:**
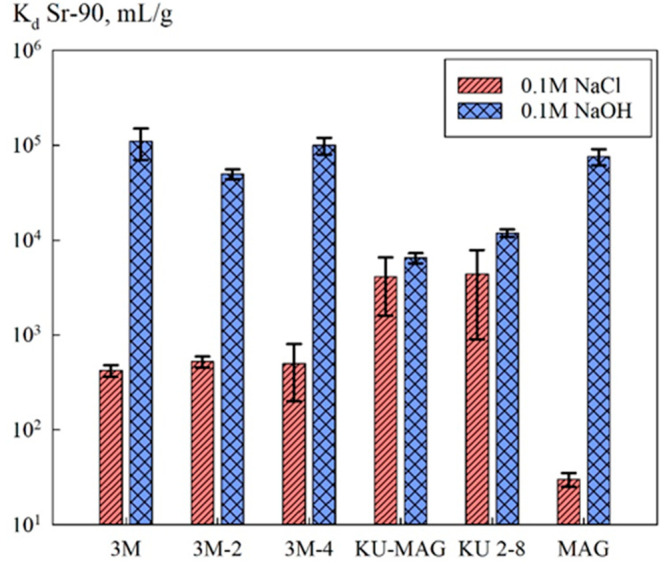
Sorption-selective characteristics of the sorbents.

Figure 6 shows the Sr-90 distribution coefficients obtained for the investigated sorption materials. Removal of the radionuclide was performed from NaCl and NaOH solutions of concentrations of 0.1 mol/L. In general, the efficiency of the sorbents was determined by the method of their synthesis, as well as by the pH value of the solution.

## 3. Discussion

In the context of the synthesis of magnetic particles of iron oxides in a particular polymer matrix, it was necessary to consider the features of the matrices mentioned above. One of the advantages of the methods for preparing magnetic composites involving chitosan was simplicity of the synthesis of iron oxide particles with chitosan by the method of co-precipitation.

Unlike the XRD image of the KU-MAG resin filled with magnetite, for which a broad diffuse peak characteristic of the KU-2-8 polymer is observed in the 2-theta angle range from 10 to 30 degrees KU-2–8 (Figure 3, curve 2), the image of chitosan filled with iron oxide does not contain peaks inherent to chitosan itself that indicates biopolymer amorphization. The latter simplifies the analysis of iron oxide phases in the composite. The X-ray diffraction analysis data showed that crystalline compounds were formed in the chitosan matrix. Simultaneously with the precipitation of chitosan, phases of Fe_3_O_4_ magnetite and FeO(OH) goethite were formed. The size and, consequently, the surface area of iron oxides are known to depend on the conditions of crystals formation and growth. The calculated particle sizes are shown in Table 1. Under the conditions of formation in the polysaccharide matrix, the particle size in the magnetic sorbent 3M equaled 9.7 nm, whereas in the sorbent 3M-2 broadening of the peak at 2θ 20.9° on the X-ray pattern indicated a decrease of the particle size (Figure 2, Table 1) down to 5.9 nm.

In the presence of chitosan under hydrothermal conditions at a temperature of 175 °C, magnetite particles of a size of 27.8 nm were formed; however, the thermal destruction of chitosan occurred at the same time, which excluded the possibility of the production of a composite sorbent. The thermal decomposition of chitosan resulted in a mineral residue in the form of silicon carbide. Since the styrene divinyl-benzene matrix was stable under hydrothermal conditions, it was possible to prepare a composite with particles of a size of 9.7 nm.

According to the provided data (Figure 4), all the studied composite materials were magnetically soft (H_c_ ≤ 50 e), with saturation magnetization values depending on the iron content in the sample. A distinctive feature of the curve of the sample with the lowest iron content consisted of the absence of saturation even under the external field voltage of 10 kOe. Such a response of the magnetization dependence curve to the external field of the sample with the lowest iron content indicated that this material contained, in addition to sufficiently large particles with ferromagnetic ordering at room temperature, particles of sizes smaller than the critical one (for Fe_3_O_4_ d ≤ 128 [23]), which manifested superparamagnetic properties.

Figure 6 shows the sorption-selective characteristics of the sorption materials. In general, the efficiency of the radionuclide removal in alkaline media increases substantially, which is related to the formation of strontium complexes [17]. Due to low mineralization, in the case of sorbents KU-MAG and KU 2-8, sorption proceeded mainly on the functional groups of the ion-exchanger [21]. It is worth mentioning that for chitosan-based composites in an alkaline media, while the Sr-90 distribution coefficients remained at the same level along with the decrease of the mass fraction of the inorganic phase. This fact was presumably related to an increase in the accessibility of the sorption sites of magnetite due to the decrease of the particle size. Here, the efficiency of the radionuclide extraction by the chitosan sorbents was comparable to that of the pure magnetite powder.

An interesting feature is concerned with the increase of the values of *G_max_*, as well as *K_L_* and *K_LF_*, and the respective decrease of the iron oxide mass fraction at the transition from the 3M sorbent to 3M-4. The latter corroborates the assumption on the accessibility of sorption sites, likely because of the increase of the surface area of the inorganic phase. Furthermore, the change of the sorbent morphology from the monolith (3M) to the sponge-like (3M-4) one ensures high accessibility of sorption sites.

## 4. Materials and Methods

Chitosan was purchased from AO Vostok-Bor (Dal’negorsk, Russia); the degree of acetylation was 0.25, and the viscosity-averaged molecular weight was 250 kDa. Iron(III) chloride (FeCl_3_ × 6H_2_O), iron(II) sulphate (FeSO_4_ × 7H_2_O), ammonium hydroxide (NH_4_OH), hydrochloric acid (HCl), strontium (stable) chloride (SrCl_2_ × 6H_2_O), and sodium hydroxide (NaOH) were purchased from Nevareaktiv (Saint Petersburg, Russia). All chemicals were of the analytical grade and used as received without additional purification.

The synthesis of the composite sorbent based on chitosan (3M) was carried out as follows. A solution containing 0.036 mol of Fe(II) and 0.018 mol of Fe(III) of a volume of 50 mL was added to a 1% chitosan solution of a volume of 300 mL. A solution of NH_4_OH of a concentration of 0.5 mol/L was added dropwise to the resulting mixture until a slightly alkaline reaction (pH 8). The resulting precipitate was filtered under vacuum, washed with distilled water, and dried in air for 48 h. The dried material was additionally heated at 105 °C for 6 h, after which it was ground, and a fraction of 0.1–0.2 mm was isolated. The synthesis of the 3M-2 sorbent was performed in a similar way; 50 mL of a solution containing 0.018 mol of Fe(II) and 0.009 mol of Fe(III) was added to 300 mL of a 1% chitosan solution under constant stirring. The obtained mixture was added dropwise with a NH_4_OH solution of a concentration of 0.5 mol/L until a weakly alkaline reaction (pH 8) occurred. The obtained precipitate was filtered, dried, and ground.

To synthesize the 3M-4 sorbent, 50 mL of a solution containing 0.009 mol of Fe(II) and 0.0045 mol of Fe(III) mixed with 300 mL of a 1% chitosan solution was used. The obtained mixture was added dropwise with a NH_4_OH solution of a concentration of 0.5 mol/L until a weakly alkaline reaction (pH 8) occurred. The obtained precipitate was filtered, dried, and ground.

Synthesis of the sample marked as MAG was carried out in a similar way as the synthesis of 3M, with the difference being that the precipitate additionally underwent hydrothermal treatment. The wet precipitate was transferred to a Teflon autoclave glass and filled with 50 mL of NH_4_OH solution of a concentration of 0.1 mol/L. The hydrothermal treatment was carried out for 24 h at a temperature of 175 °C, after which the reactor was gradually cooled down to room temperature. The resulting black precipitate was washed with distilled water in a Buchner funnel and dried at 85 °C until constant weight.

The synthesis of the KU 2-8 resin modified with magnetite (sample KU-MAG) was carried out according to the method suggested in [36]. Wet KU 2-8 resin in the H-form was brought into contact with a solution containing 5 × 10^−3^ mol Fe(II) and 2.5 × 10^−3^ mol Fe(III) for 12 h. Thereafter, the resin was separated from the solution, washed with distilled water, and treated with NH_4_OH solution. Then, the resin was washed from magnetite particles formed outside the resin bulk by means of decantation and transferred to the Teflon autoclave glass. A solution of NH_4_OH with pH 11–14 was added to the resin and autoclaved for 12 h at a temperature of 175 °C. The resulting resin had a black color.

Markings of the sorbents and their characteristics are provided in Table 4. The KU-Mag sorbent exhibited the lowest content of the mineral phase, which was related to a limited number of sorption sites.

Prior to the experiments, the model solutions were labeled with the Sr-90 radionuclide (1000 Bq/mL). The removal of strontium was performed under static conditions when the sorbent was continuously stirred with a model solution in a 10 mL polypropylene cylinder at a rate of 20–30 rpm in a vertical rotary shaker; the stirring proceeded for 7 days. Three parallel samples were used for each experiment, including a test experiment with no sorbent. After the specified time, the model solution was separated from the sorbent by centrifugation at a rate of 4500 rpm for 15 min, after which the residual activity was determined.

The distribution coefficient for Sr was calculated according to the following formula (1):

(1) $$K_{d}=\frac{A_{0}-A_{eq}}{A_{eq}}\times \frac{V_{st}}{m}$$

where *A_0_* is the initial activity of the model solution (Bq/mL), *A_eq_* is the equilibrium activity of the liquid medium (Bq/mL), *V_st_* is the volume of the liquid medium during sorption under static conditions (mL), and *m* is the mass of the sorbent sample (g).

To describe sorption isotherms, standard equations of Freundlich (3), Langmuir (4), and the combined Langmuir–Freundlich (L–F) Equation (5) were used.

(2) $$\Gamma \;=K_{F}\times C^{m}$$

(3) $$\Gamma \;=G_{max}\times \frac{K_{L}\times C}{1+K_{L\;}\times C}$$

(4) $$\Gamma \;=G_{max}\times \frac{K_{LF}\times C^{m}}{1+K_{LF}\times C^{m}}$$

where *G_max_* is sorption capacity (mmol/g), *C* is equilibrium concentration of Sr (mmol/L), *K_F_* is Freundlich’s constant, characterizing the adsorption ability and representing the adsorption value at equilibrium concentration equal to 1; *K_L_* and *K_LF_* are constants of adsorption equilibrium characterizing adsorbent–adsorbate binding energy; *m* is a parameter of sorption site irregularity, characterizing the change of the heat of adsorption depending on the fractional occupancy of the sorption sites.

The activity of the model solutions with respect to Sr-90 was determined using a liquid scintillation alpha-beta radiometer of the spectrometric Tri-Carb 2910 TR device (Perkin Elmer, Norwalk, CT, USA). The X-ray diffraction analysis was performed using a D8 ADVANCE diffractometer (Bruker AXS GmbH, Karlsruhe, Germany); X-ray patterns of the samples were recorded within the angle range 2θ from 3° to 85° with an increment step of 0.02° at a count of 0.6 s. Identification of the phase composition was performed using the QualX software (version 2.24) and the Crystallography Open Database (27 June 2019). Surface structure images of the investigated materials were obtained by means of scanning electron microscopy on a Carl Zeiss CrossBeam XB 1540 (Oberkochen, Germany) with an attachment for energy dispersive analysis. The results were processed using SciDavis software (version 1.23).

The size (*D*) of iron oxide particles in the original powder and in composites was determined from the XRD data using the Scherrer Equation (5):

(5) $$D=\frac{0.94\lambda }{\beta_{1/2}COS\theta }$$

where β_1/2_ is the line broadening in radians, θ is the Bragg angle, and λ is the X-ray wavelength (CuK-α 1.5406 Å).

Magnetization of the samples was measured using an MPMS XL-7 SQUID Quantum Design (San Diego, CA, USA) magnetometer in the field range of ±10,000 Oe at a temperature of 300 K. A measurement step was 100 Oe in the range of 0 to ±2000 Oe and to 500 Oe in the range of ±2000 to ±10,000 Oe.

## 5. Conclusions

The creation of composite materials based on several components serves as a platform for the development of advanced technologies for water treatment. Creation of macrosystems with the introduction of nanosized particles, i.e., the development of nanocomposite materials acquiring the advantages of both hosts and immobilized functional particles, represents a promising approach. Hosts (minerals, silicon oxides, activated carbons, polymers, and biopolymers) improve the dispersion and stability of the introduced (loaded) nanoparticles. They can facilitate the transfer or diffusion of the contaminant to the matrix-filling particles (loaded particles). Host matrices most likely prevent the release of guest nanoparticles into the treated solutions. Therefore, it is possible to combine nanocomposites with existing water treatment technologies using nanosystems in boiling bed devices or columns.

In the future, the use of magnetic sorbents will allow the development of more sustainable technologies for water decontamination and the extraction of valuable components from solutions.

## Figures and Tables

**Table 1:** Particle sizes of iron oxides in the investigated sorption materials.

Samples	Sizes (nm) for 2θ Angles										
	20.3	20.9	21.5	30.2	30.3	34.5	35.5	35.6	35.3	35.6	35.9
M	9.7						7.9				
3M-2	5.9						6.4				
3M-4	12						5.2				
MAG				27.8				25.2			
KU-MAG								9.7			

**Table 2:** Results of estimation of the stability of composite sorbents in NaCl solution (0.1 mol/L).

Sorbent	Washed-Out Iron Mass (mg) *	Washed-Out Iron Fraction (%) **
3M	0.1	0.02
3M-2	0.08	0.04
3M-4	0.18	0.17
KU-MAG	0.12	0.23

* Recalculated to composite mass; ** Recalculated to mass fraction of inorganic phase.

**Table 3:** Parameters of Freundlich, Langmuir, and Sips equations for the isotherms of Sr^2+^ sorption on virgin composite sorbents.

Sorbent	Freundlich Equation		Langmuir Equation			Sips Equation				
	*K_F_*	*n*	*R^2^*	*K_L_*	*G_max_*	*R^2^*	*K_LF_*	*G_max_*	*n*	*R^2^*
**3M**	0.002 ± 0.0002	0.488 ± 0.054	0.972	0.37 ± 0.05	0.009 ± 0.001	0.993	0.34 ± 0.08	0.009 ± 0.002	0.92 ± 0.15	0.994
**3M-2**	0.004 ± 0.0004	0.564 ± 0.064	0.973	0.26 ± 0.02	0.019 ± 0.001	0.998	0.017 ± 0.001	0.28 ± 0.03	1.1 ± 0.1	0.998
**3M-4**	0.005 ± 0.001	0.606 ± 0.044	0.989	0.18 ± 0.03	0.029 ± 0.003	0.993	0.14 ± 0.06	0.039 ± 0.016	0.85 ± 0.15	0.994
**KU-MAG**	0.161 ± 0.001	0.443 ± 0.007	0.999	1.39 ± 0.47	0.31 ± 0.04	0.975	0.004 ± 0.075	43 ± 85	0.45 ± 0.0291	0.999

**Table 4:** Markings and characteristics of the sorbents.

Sorbent	Matrix	Content of Inorganic Phase (wt. %)
3M	Chitosan	42
3M-2	Chitosan	21
3M-4	Chitosan	10.5
KU-MAG	Styrene divinyl-benzene	5.3
KU 2-8	Styrene divinyl-benzene	-
MAG	-	1000
